# High throughput nonparametric probability density estimation

**DOI:** 10.1371/journal.pone.0196937

**Published:** 2018-05-11

**Authors:** Jenny Farmer, Donald Jacobs

**Affiliations:** 1 Department of Physics and Optical Science, University of North Carolina at Charlotte, Charlotte, NC, United States of America; 2 Department of Bioinformatics and Genomics, University of North Carolina at Charlotte, Charlotte, NC, United States of America; 3 Center for Biomedical Engineering and Science, University of North Carolina at Charlotte, Charlotte, NC, United States of America; University of California Irvine, UNITED STATES

## Abstract

In high throughput applications, such as those found in bioinformatics and finance, it is important to determine accurate probability distribution functions despite only minimal information about data characteristics, and without using human subjectivity. Such an automated process for univariate data is implemented to achieve this goal by merging the maximum entropy method with single order statistics and maximum likelihood. The only required properties of the random variables are that they are continuous and that they are, or can be approximated as, independent and identically distributed. A quasi-log-likelihood function based on single order statistics for sampled uniform random data is used to empirically construct a sample size invariant universal scoring function. Then a probability density estimate is determined by iteratively improving trial cumulative distribution functions, where better estimates are quantified by the scoring function that identifies atypical fluctuations. This criterion resists under and over fitting data as an alternative to employing the Bayesian or Akaike information criterion. Multiple estimates for the probability density reflect uncertainties due to statistical fluctuations in random samples. Scaled quantile residual plots are also introduced as an effective diagnostic to visualize the quality of the estimated probability densities. Benchmark tests show that estimates for the probability density function (PDF) converge to the true PDF as sample size increases on particularly difficult test probability densities that include cases with discontinuities, multi-resolution scales, heavy tails, and singularities. These results indicate the method has general applicability for high throughput statistical inference.

## Introduction

A central tenant of statistical modeling is that different samples of a random process will exhibit varying outcomes that are quantified by probabilities. For continuous random variables, a probability density function (PDF) assigns a probability for observing a value that falls within a certain range of interest. How to empirically determine a PDF that is consistent with *N* samples of univariate data is a well-studied problem in mathematics, and its practical significance is ubiquitous. For example, applications are found across a broad range of fields such as in astronomy [1–3], geoscience [4, 5], climate change [6], bioinformatics [7–10], medicine [11–13], and engineering [14–17]. Additionally, PDF estimation is crucial in areas such as finance and economics [18–21], and continues to be relevant in new areas of mathematics and information science [22–24].

Many estimation methods have been successfully employed to fit a random data sample to parameters of a known functional form. However, it is often the case that the functional form that describes the underlying random process is unknown. In these situations, a particular functional form is often considered out of convenience, especially when there is limited data. When the data has specific features, it is common to employ a mixture model involving a linear superposition of known functional forms. In all these cases expert knowledge is required to proceed. More generally, nonparametric methods are available to estimate a PDF when a parameterized functional form for the PDF is unknown. Nonparametric estimators make minimal assumptions and allow data characteristics alone to determine the form of the distribution [25–27] from which statistical inferences can be made. As such, nonparametric methods are rapidly growing in relevance along with the emerging field of data driven decision-making [28–30].

The simple nonparametric method of binning the data to obtain a histogram offers insight into how the data is distributed, but this approach falls short of producing an analytical function to represent the probability density. A problem encountered with binning data is in the choice of a bin width, which dramatically affects the appearance of the histogram [31–36]. Kernel density estimation (KDE) is a common approach that replaces a histogram with a smooth function that represents a consensus of the data points that fall within local binned regions. In practice, KDE constructs a smooth curve to track the shape of a histogram [37] using a linear combination of kernel functions. The kernel functions are usually selected to be Gaussian distributions. Unfortunately, when bins of random samples are smoothed out in this way, differentiating between fluctuations in a random sample versus sharp features in the true PDF is challenging. If multiple resolution scales are present (e.g. multiple modes with different variances) it is particularly difficult to smooth out unwanted noise while retaining actual features, and it is also difficult to accurately account for sharp boundary conditions.

To address these difficulties, advanced approaches are available to adapt to multiple resolution scales [38–41] and to employ alternate kernel functions [42], in part to handle boundary effects [43]. However, relying on one’s intuition about data characteristics and conditions on the smoothness of an unknown PDF remains a drawback. Furthermore, KDE is intrinsically limited because it produces a result that is a convolution of the PDF with the selected kernel function employed to smooth the data [25]. In practice the effect of the convolution is the welcomed smoothness of KDE estimates, which is ultimately controlled by user intuition. In spite of its simplicity and utility, KDE frequently does not give an accurate representation for heavy tail(s) or sharp boundaries of a PDF [26].

The maximum entropy method (MEM) [44–46] is another approach for estimating a PDF, providing an analytical form described by *p*(*v*) on support [*a*,*b*]. Certain known constraints are expressed in terms of moments for a set of functions {*g*_*j*_(*v*)} that are bounded to ensure all moments exist. As a function of *p*(*v*), the functional $\tilde{S}$ comprised of the entropy, *S*, with any number of desirable constraint conditions added is written as:

$$\tilde{S}\left[p\left(\nu \right)\right]=\lambda_{0}\left(\int p\left(\nu \right)d\nu -1\right)+\sum_{j=1}^{D}\lambda_{j}\left(\int g_{j}\left(\nu \right)p\left(\nu \right)d\nu -\langle g_{j}\rangle \right)-\int ln\left[p\left(\nu \right)\right]p\left(\nu \right)d\nu$$ (1)

The variables, *λ*_*j*_, are Lagrange multipliers that play an important role in determining the shape of *p*(*v*) based on knowing the exact population averages {〈g_*j*_〉} for which the *λ*_*j*_ are conjugate to. In the process of maximizing entropy, the −ln[*p*(*v*)]*p*(*v*) term in the integrand constrains all viable *p*(*v*) functions to be non-negative. The solution to Eq (1) for *p*(*v*) is straightforward to obtain [47], which works out to be

$$p\left(\nu \right)=exp\lfloor \left(\lambda_{0}-1\right)+\sum_{j=1}^{D}\lambda_{j}g_{j}\left(\nu \right)\rfloor .$$ (2)

The Lagrange multiplier, *λ*_*o*_, is adjusted to normalize the PDF. Although very powerful in principle, a limitation with traditional MEM is that it is formulated as a parametric model [5], where the number of moment constraints is known in advance in the sense that a priori knowledge of {〈g_*j*_〉} is required and specific to the problem of interest.

Within a nonparametric framework, the best form of the constraint functions and the number of these functions to consider is not known in advance. Consequently, the set of {g_*j*_} should form a complete set of orthogonal functions and, to guarantee converge at all orders, |*g*_*j*_(*v*)| must be bounded [47]. As higher orders of g_*j*_ are included in the series expansion, sharper features in the PDF are captured because higher order orthogonal functions oscillate more rapidly. The critical problem we address in this paper is how to determine the number of functions to include in the series expansion that sits within the exponential in a way that resists over fitting to the data using objective criteria. One approach to this problem applies the method of maximum likelihood to the exponential form of the PDF from MEM given in Eq (2) with supplemental Bayesian or Akaike information criterion [21, 48]. However, these methods are based on relative measures and not based on an absolute scale, which we develop here.

To facilitate high throughput analysis, we report a novel method based on MEM to estimate probability densities while satisfying five essential elements for the modern era of data science. First, the method is nonparametric because data characteristics will be largely unknown other than that the random variables can be modeled as continuous, and they are, or can be approximated as, independent and identically distributed. Second, a universal scoring function is employed as an absolute metric to test the quality of the estimated probability density function (PDF) to eliminate subjective decisions such as how smooth the PDF should be. Third, the method is data-driven such that the resolution of features in the PDF improves commensurately with the number of observations in a statistical sample. Forth, the estimated PDF is expressed as an analytical function to facilitate subsequent statistical analysis. Fifth, the process is fully automated with an almost nonexistent failure rate, and, if the procedure fails, an error message will give diagnostics about why.

These aspects, combined with computational efficiency, have resulted in an effective method we now routinely employ for structural bioinformatics analyses [49]. A typical application involves determination of PDFs for many thousands of samples classified by conditional requirements, and for each case there are thousands of data items per sample. The scope and comparative nature of this type of application makes standard kernel density estimation practically prohibitive because the properties of these distributions are not known in advance. The objective of this work is to describe and benchmark the method. In addition, despite the need for automation, scaled quantile residual plots are introduced as a powerful visualization scheme for human assessment of the quality of the solutions in great detail.

The rest of this paper is organized as follows. In Section 2, the nonparametric MEM is described, and the algorithm defined. Also, new statistical metrics we use to monitor the quality of PDF estimates are defined. In Section 3, properties of SOS are employed to construct a scoring function based on a quasi-log-likelihood function for SURD. A key result is that this scoring function is empirically found by numerical experiments to be sample size invariant. In Section 4, benchmark results for five example distributions that embody difficult facets are discussed. In Section 5, conclusions are made based on the numerical results that demonstrate the new nonparametric method is robust, versatile, and consistent with statistical resolution. Further implementation details and additional examples are archived on the arXiv web server [50].

## Methods

### 2.1 Nonparametric maximum entropy method

The Lagrange multipliers that appear in Eq (2) define a point in parameter space, which is denoted here as ***λ***. At different points in parameter space the shape of the PDF changes. Starting at ***λ***^(0)^ (some initial guess), the algorithm makes trial random steps in parameter space that land at a new ***λ***. A trial step produces an estimate *p*_*e*_(*x*|***λ***) that may have a functional form farther or closer to the true PDF, *p*(*x*), which is measured by a scoring function. The scoring function does not require *p*(*x*) to be known. The trial step is taken when the score improves; otherwise the trial step is not taken. Labeling only the successful steps, ***λ***^(*i*)^ indicates that *p*_*e*_(*x*|***λ***^(*i*)^) is closer to *p*(*x*) than *p*_*e*_(*x*|***λ***^(*i*−1)^). Random trials are generated using the same random search method that was employed previously [47]. The novel contribution of this work replaces minimization of least squares error between calculated and empirical moments using a plugin method by iteratively improving the universal scoring function until a target value is reached.

The critical improvement for the new MEM is that the probability for a certain error between a trial PDF and the true PDF is calculated using a universal statistical metric for *sampled uniform random data* (SURD) on the interval [0,1] without knowing *p*(*x*). The word “universal” emphasizes that this scoring function is problem independent, and approximately sample size independent. The strategy employed here transforms random variables {*V*_*k*_} onto new random variables {*U*_*k*_} that span the interval [0,1] using the cumulative distribution function (CDF) associated with a trial PDF. A trial PDF is considered an appropriate approximation to the true PDF when {*U*_*k*_} exhibits the same statistical properties as that of SURD. This is done by iteratively adjusting Lagrange multipliers to improve the match between the statistics of {*U*_*k*_} and SURD as quantified by the scoring function (defined in section 3). Importantly, as the procedure is iterated the parameter space dimension is increased as needed.

### 2.2 Bound intervals and censor windowing

When working with numerical estimates, describing a PDF on a bound interval [*a*,*b*] does not pose limitation in practice. First, if bounds on the random variables are known, they should be used to obtain maximum accuracy for the model PDF. Second, selecting a finite domain is necessary in applications where samples are censored by windowing. For example, a selection rule may measure the value of a random variable *V*_*k*_ within the limits [*a*,*b*], while counting the number of events when *V*_*k*_ falls outside this range. The ratio, *R*_*ab*_, of the number of events measured within the range [*a*,*b*] to the total number of events gives an estimate for the probability, $P_{ab}=\int_{a}^{b}p(v)\;dv$. The normalization constraint required in the MEM generalizes to *P*_*ab*_ = *R*_*ab*_, where uncertainty comes from the empirical estimate for *P*_*ab*_. The location of the interval [*a*,*b*] is not critical because a model PDF can be accurately calculated within the censoring window regardless of how much data is discarded, provided the amount of discarded data is counted.

In applications without a priori knowledge of the limits on *V*_*k*_, we use data-driven criteria involving two conditions to define a censor window [*a*,*b*]. For a sample of *N* data items sorted from lowest to highest values, we define *a*_1_ = *V*^(1)^ − [*V*^(5)^ − *V*^(1)^] and *b*_1_ = *V*^(*N*)^ + [*V*^(*N*)^ − *V*^(*N*−5)^]. Here, the sort index is used as a superscript. These limits include all sampled data. When the true PDF has a heavy tail due to extreme statistics, this range must be reduced to perform accurate numerical integration for the CDF in practical compute times. A balance is reached that removes extreme events to ensure numerical integration is tractable yet strives for the largest possible censor window. To balance these two disparate objectives, extreme values are identified according to the formulas: *a*_2_ = *Q*_25_ − *c*(*Q*_75_ − *Q*_25_) and *b*_2_ = *Q*_75_ + *c*(*Q*_75_ − *Q*_25_) where *Q*_25_ and *Q*_75_ are the 25% and 75% quantiles respectively. When *c* = 3, the outliers identified have been called *extreme* [51, 52]. For this work, *very extreme* values are identified using *c* = 7. The interval [*a*,*b*] where *a* = max(*a*_1_, *a*_2_) and *b* = min(*b*_1_, *b*_2_) sets a boundary of the censor window whenever it is unknown. Note that power law tails can be identified accurately within a censor window. This data-driven process of defining the censor window requires no a priori knowledge of data characteristics. In certain extreme tests, we have set the value of *c* to be over 25, but the computation time slows down.

Without loss of generality, a linear transformation is employed to map the random variable *V*_*k*_ to *X*_*k*_ according to $X_{k}=\frac{2V_{k}-b-a}{b-a}$. This transformation conveniently maps the domain onto the interval [−1,1], which is a natural range for Chebyshev polynomials [53]. In particular, Chebyshev polynomials of the first kind are level-functions, because |T_*j*_(*x*)| ≤1 for −1 ≤ *x* ≤ 1 for all *j*. Importantly, they form a complete orthogonal basis on [−1,1]. The Chebyshev polynomials have been selected to construct the PDF in the form shown in Eq (2) because of convenient properties. After mapping the random variables {*V*_*k*_} onto {*X*_*k*_}, the algorithm next maps {*X*_*k*_} onto new random variables {*U*_*k*_} that span the interval [0,1].

### 2.3 Implemented algorithm for random search

**Fig 1:**
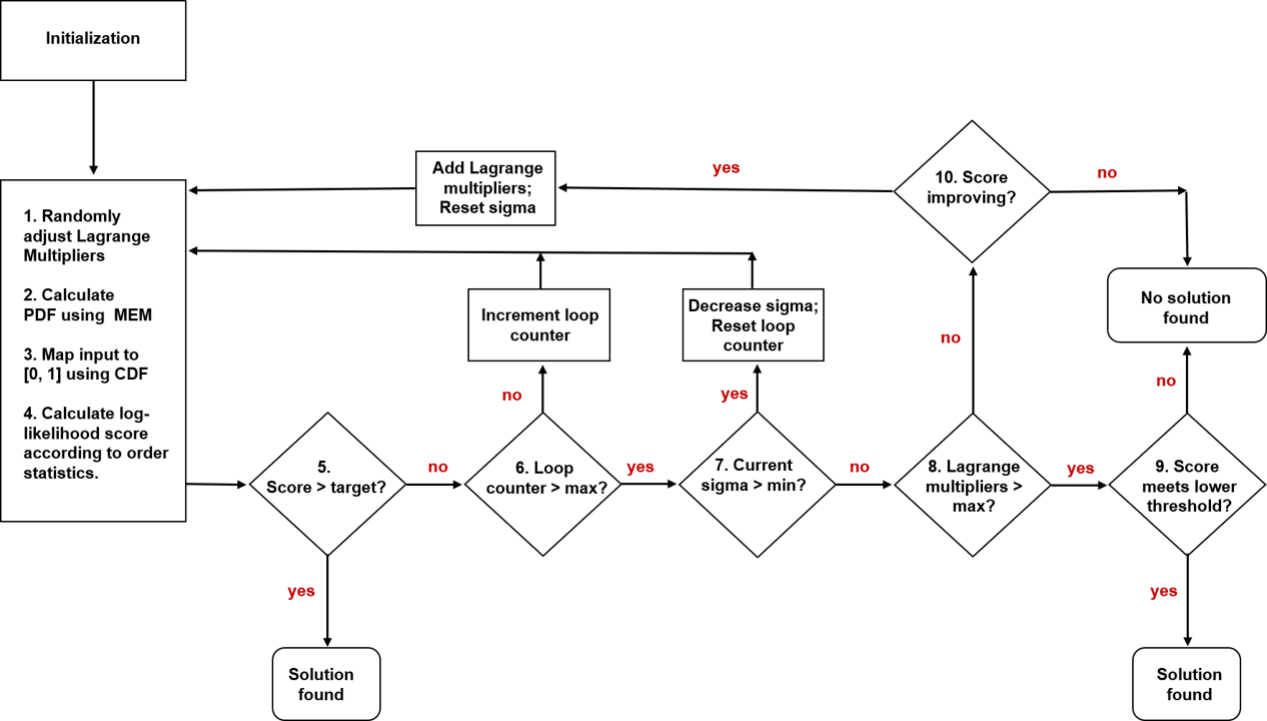
Flow chart for main loop. Iterative flow summarizing major steps and sub-loops. The numbers on procedures and decisions correspond to the pseudo-code for the main loop.

At a high level, Fig 1 shows the processing flow of the implemented algorithm. The basic idea is to reach a target score with the minimum number of Lagrange multipliers possible. Some of the important characteristics of the flowchart are summarized here. First, step 2 requires the greatest number of calculations as it constructs the PDF from the given set of Lagrange multipliers and then uses adaptive integration to calculate the corresponding CDF. Second, the decision steps 6 and 7 are part of a random search method, where each step in the parameter space of Lagrange multipliers is randomly selected using a Gaussian distribution characterized by the sigma-parameter, which sets the standard deviation of the step size. The sigma-parameter starts high and is then gradually decreased until a minimum step size is reached, corresponding to a random walker moving down a funnel. For this reason, we refer to this random search method as funnel diffusion [54]. Third, if the target score is reached, the program finds a solution (decision step 5) and terminates. Fourth, if the score is improving but the target score is not yet reached after funnel diffusion terminates (decision step 7), then more Lagrange multipliers are added and, the random search is restarted again with the goal to eventually reach the target score. Finally, if the target score is not reached before a maximum number of Lagrange multipliers are tried (decision step 8), or if adding more Lagrange multipliers do not significantly better the score (decision step 10), then further attempts to better the score are terminated. However, if the current score, albeit below the target score, is above a minimum acceptable level (decision step 9) then a solution is found. The formula for how to calculate the score and how the universal scoring function is determined is discussed in section 3. Furthermore, the utility and significance of the target and minimum acceptable scores will be discussed in detail at the end of section 3 in relation to our key results involving the universal scoring function.

The steps listed below in the pseudo-code explain in more detail how PDF-trials are explored and, together with a scoring function, a series of successively improved PDF-models are generated until convergence criteria are met. Each of the three main sections (initialization, main loop, and end processing) are executed a specified number of times to produce an ensemble of viable PDF models that account for expected levels of fluctuations in finite size samples. Over an ensemble of possible PDF models, for example we usually consider 5 PDF models per sample, model selection is the next step as explained in the next subsection.

#### Initialization steps

1. Initialize one Lagrange multiplier with a value of zero.
2. Create the PDF for a uniform distribution. Calculate and normalize the CDF. Set the first Lagrange multiplier as the normalization constant.
3. Map transformed sample data to [0,1] using the CDF.
4. Using single order statistics, create a log-likelihood score for mapped data
5. If log-likelihood score is consistent with SURD within user-defined target, end program and accept uniform distribution as a solution (default target is 40%).
6. Add an additional Lagrange multiplier and initialize it to zero
7. Set current funnel diffusion step size, sigma, to user-defined value (default is 0.1)
8. Set the funnel diffusion decay_rate to user-defined value (default is $\frac{\sqrt{2}}{2}$), such that sigma → decay_rate × sigma.
9. Set final allowed step size to user-defined value (default is 0.001).
10. Initialize funnel-step loop counter to zero

#### Main loop

1. Execute a random search method on Lagrange multipliers by making a random step using a Gaussian distribution from each of the current values, using the current sigma as the standard deviation
2. Create a PDF using new Lagrange multipliers. Calculate and normalize the CDF. Set the first Lagrange multiplier as the normalization constant.
3. Map transformed sample data to [0,1] using cdf
4. Using order statistics, create a log-likelihood score for mapped data
5. If log-likelihood score is consistent with SURD within defined target, accept current PDF as the solution and go to step 11, else continue to step 6. Scoring procedure is described in section 3.
6. If random search loop counter < user-defined maximum, increment loop counter by one, and go to step 1 (default for max loop counter is *F*_*m*_ = 100), else continue to step 7
7. If current sigma is > final allowed step size, decrease current sigma by decay_rate, reset loop counter to zero, and go to step 1, else continue to step 8
8. If number of Lagrange multipliers < maximum number allowed (default max = 300), go to step 10, else continue to step 9
9. Reassess current log-likelihood score to determine if it meets a minimum SURD threshold of 5%. If the score meets this lower threshold, tag this solution as a success, else tag solution as a failure and report output for diagnostics. Go to step 11.
10. If score has not improved by a minimum (user-defined) percentage in a user-defined number of consecutive Lagrange multiplier additions (defaults are 1% and 3, respectively) tag solution as a failure, report output for diagnostics, and go to step 11, else add an additional two Lagrange multipliers, initialize them to zero, increment loop counter by one, and go to step 1.
11. End program

#### End processing

1. Apply the Lagrange multipliers on raw input data to create PDF and normalized CDF for final solution.
2. Write out full solution to a file, even if it was a failure, including all parameters, score, and raw data with PDF and CDF

### 2.4 Model selection

From this data-driven iterative random search procedure, an ensemble of possible PDF model solutions is generated, from which an estimate is made, and uncertainties can be quantified. Although uncertainties in KDE estimates can be quantified [55], in practice it is usually overlooked and under-appreciated. With an ensemble of possible PDF models that fit the data (but with different scores) there are numerous ways to implement model selection. Here, we considered a simple approach that allows us to benchmark the method carefully by checking the model solutions of all members of the ensemble. Following the algorithm for the random search method given above, different model PDF solutions are obtained for the set target of the scoring function per run. For *M* different models (typically we generate 5) the most central PDF model among the set is selected as the estimate. To identify which PDF model should be used as the estimate, the total squared error between each PDF model to all other PDF models within the ensemble is calculated. Specifically, *M*(*M* − 1) calculations are performed to arrive at *M* total squares error, one per PDF model. The PDF model that has the least squares error is selected to be the estimate (i.e. the most central). Error bars are readily obtained from this data as well. We mention that the random search method can be replaced with a Monte Carlo method that takes an equilibrium ensemble average with acceptance probabilities related to the universal scoring function. In this case, no target score or minimum score would be needed, but the criterion for when equilibrium is achieved would need to be established, and this approach is left for future work.

### 2.5 Order statistics and scaled quantile residual plots

The random data {*U*_*k*_} is sorted from smallest to largest values. The sorted data is labeled as {*U*^(*s*)^} where *s* is a sorting index from 1 to *N*, such that *U*^(s)^ < *U*^(*s*+1)^ for all *s*. The properties of order statistics for a uniform probability distribution are well known in the literature [7, 56, 57]. Employing single order statistics on *N* observations, the probability density for finding *U*^(*s*)^ at *u*, is given by

$$p_{s}(u|N)=\frac{N!{\left(1-u\right)}^{N-s}u^{s-1}}{(N-s)!(s-1)!}$$ (3)

From Eq (3) it follows that the mean position for *U*^(*s*)^ and its variance are respectively given by

$$u(s|N)=\frac{s}{N+1}\;\sigma^{2}=\frac{s(N+1-s)}{(N+2){\left(N+1\right)}^{2}}.$$ (4)

After simplifying, the standard deviation characterizing typical fluctuations in the position of *U*^(*s*)^ about its mean is given by

$$\sigma =\frac{\sqrt{u(1-u)}}{\sqrt{N+2}}.$$ (5)

Notice that typical sample size fluctuations within the set of positions {*U*^(*s*)^} about their respective average positions drop as $\frac{1}{\sqrt{N+2}}$ according to Eq (5). Therefore, larger sample sizes will resolve single order statistics better, and the size of typical fluctuations provides a good way to quantify and visualize statistical resolution.

When SURD is generated in numerical experiments, deviations of the random variables from their population average define a quantile residual. For the *s*-th sorted data point, the residual is defined by δ_*s*_ = *U*^(*s*)^ − *μ*(*s*|*N*). A quantile residual plot is then defined by plotting the residual as a function of the expected mean, *μ*(*s*|*N*). Unlike a standard QQ-plot that approaches a straight line with slope of 1 going from 0 to 1 on both the abscissa and ordinate as sample size increases [58], plotting *δ*_*s*_ as a function of *μ*_*s*_ provides a much easier way to visualize deviations away from the perfect straight line, especially for large sample sizes. Unfortunately, residual plots are not very useful either when employed to track how a model PDF converges to the true PDF as sample size increases, because residuals shrink to zero as *N* increases. Therefore, we scale the deviations by a sample size factor to arrive at a sample size invariant measure. Specifically, let

$${△}_{s}=\sqrt{N+2}\delta_{s}=\sqrt{N+2}\left(U^{\left(s\right)}-\frac{s}{N+1}\right).$$ (6)

**Fig 2:**
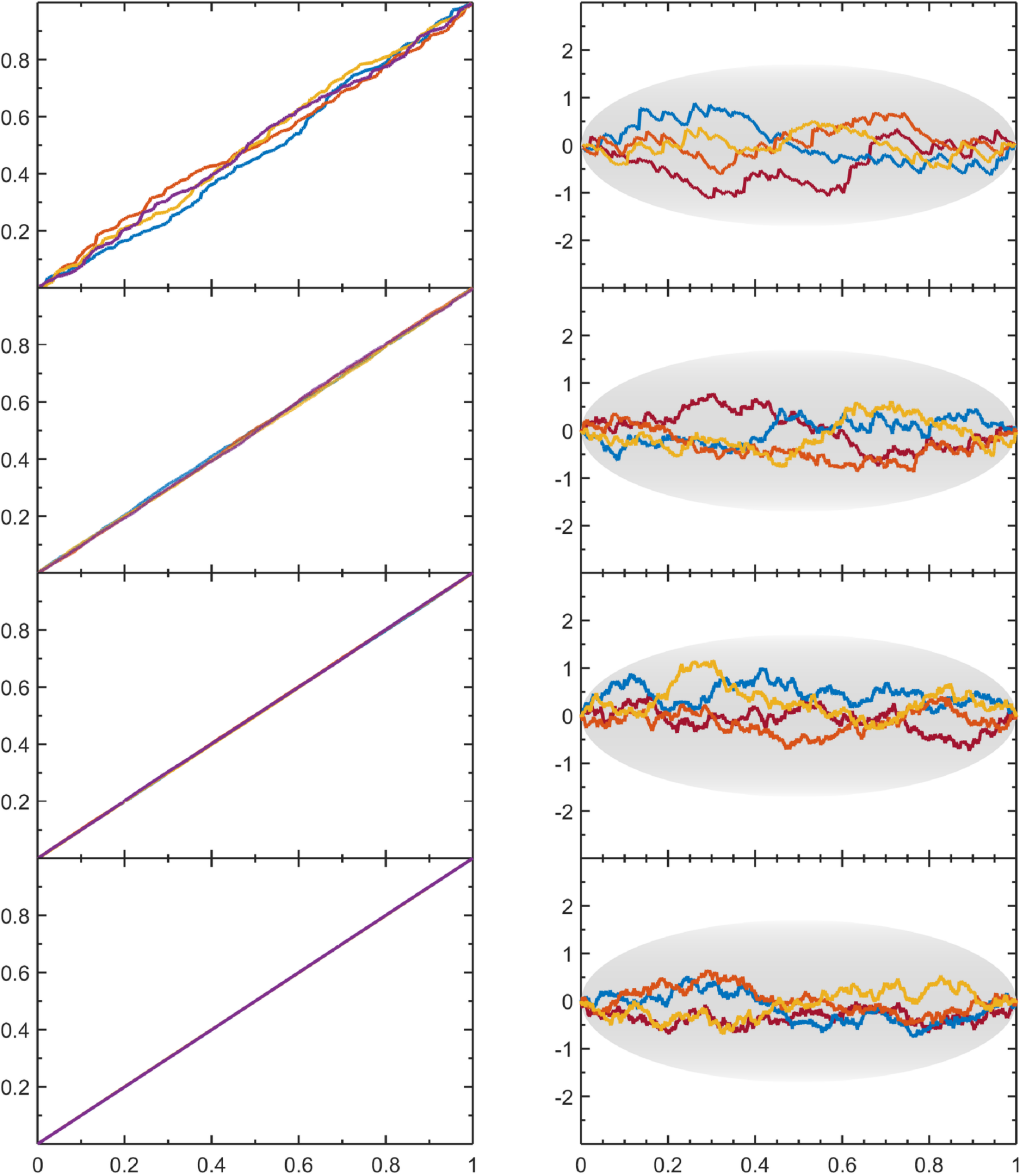
Comparing quantile plots to their scaled residuals. For SURD on [0, 1] the corresponding QQ plots (left column) and SQR plots (right column) are shown for increasing sample sizes with *N* = 2^8^, 2^12^, 2^16^, 2^20^ from top to bottom.

The variable Δ_*s*_, when plotted against *μ*(*s*|*N*), defines a scaled quantile residual plot (SQR-plot). The SQR-plot makes it easy to visually assess results for different sample sizes, and systematic errors in PDF estimates can be identified without knowing the true PDF. Using a uniform distribution on [0,1] to generate SURD, QQ-plots and SQR-plots for various sample sizes are compared in Fig 2. The SQR-plot provides a sample size independent characterization that is commensurate with the expected level of statistical resolution. In particular, the SQR-plots for SURD show typical fluctuations characterized by $\sqrt{\mu (1-\mu )}$. Importantly, typical fluctuation amplitudes are not equal across positions. The fluctuations are greatest near the center of the range and decrease towards zero at the ends due to the bounded nature of the data sample on the range [0,1]. The maximum expected fluctuations per position are approximated by the gray oval shown on each SQR-plot (Fig 2). The envelope for this gray oval is constructed by multiplying Eq (5) by $\pm 3.4\sqrt{N+2}$ to carve out a range that includes more than 3 standard deviations, corresponding to a confidence greater than 99% (confirmed by numerical experiments on simulated SURD). Therefore, deviations outside of the oval area should be suspect when evaluating SQR-plots.

### 2.6 Figure of merit

A figure of merit (FOM) is introduced to give a single number that reflects how typical the data sample is compared to samples generated by the PDF estimate. This is done through a series of brute force pairwise comparisons between generated samples from the PDF estimate to the input sample. The steps taken to calculate FOM are as follows.

1. Create 10 data samples from the CDF, sort them and use as ‘reference samples’
2. Create an additional 100 data samples from the CDF, sort them and use as ‘test samples’
3. Calculate the average difference, per position s, between the original data sample and the 100 test samples. Call this *δ*_*sample*_(*s*).
4. Calculate the average difference, per position s, between *each* of the 10 reference samples and the 100 test samples. Call this *δ*_*test*_(*s*).
5. Find the average and standard deviation, per position *s*, of the differences between each reference sample to all test samples, by comparing 100 sample sets to the r-th reference sample. Call the average deviation *δ*(*s*,*r*) and its standard deviation *σ*(*s*,*r*).
6. For each of the 10 reference samples, calculate a FOM per position.
  $$FOM_{test}(s,r)=1-\frac{\left|\delta (s,r)-\delta_{test}(s)\right|}{2*\sigma (s,r)}$$
7. Calculate a FOM per position for the original data sample.
  $${FOM}_{sample}(s,r)=1-\frac{\left|\delta (s,r)-\delta_{sample}(s)\right|}{2*\sigma (s,r)}$$
8. Calculate 〈*FOM*_*test*_(*s*,*r*)〉, which reflects an average quantity over 10 reference samples and all *k* positions. This average FOM is denoted as *FOM*_*test*_. Similarly, the standard deviation for *FOM*_*test*_ is calculated and denoted as *FOM*_*σ*_.
9. Likewise, calculate 〈*FOM*_*sample*_(*s*,*r*)〉 denoted as *FOM*_*sample*_, which is an average over all reference samples and all *k* positions.
10. Using the FOM moments calculated in steps 8 and 9, compute a single number for FOM,

$$FOM=1-\frac{\left|{FOM}_{test}-{FOM}_{sample}\right|}{2*FOM_{\sigma }}.$$

The FOM is a number on the interval (−∞,1), where a positive value indicates that the input sample has similar characteristics to a typical member in the ensemble. When FOM is positive, there is high confidence the input sample reflects a typical outcome from the estimated PDF. Moreover, a FOM > ½ indicates an excellent model.

### 2.7 Hierarchical data augmentation

Processing the sampled data hierarchically dramatically reduces computational cost. The strategy employed sorts the full set of data, {*U*^(*s*)^}, which has been transformed on the range from 0 to 1. The collection of these sorted data points has information about the global shape of the CDF that transforms *X*_*k*_ to *U*_*k*_. For example, out of 100,000 data points, the 27,000-th data point cannot be anywhere in the range from [0,1], but rather, it must be after the 26,999-th point and before the 27001-th point. Therefore, uniformly selecting a subset of 1,000 points from 100,000 points by skipping every 100 points is not equivalent to throwing out 99% of the information. Instead, the large-scale shape characteristics of the CDF are retained by a small subset of data. Sharper features are subsequently resolved as more uniformly spaced data is added. This intuitive idea is embodied in the expansion in terms of orthogonal functions, starting with those that vary slowly to those with rapid variations (wiggles).

The hierarchical process is invoked for datasets with more than 1,025 observations; otherwise all data is used. For large datasets, the first step is to define a base subset having 1,025 points. Each subset thereafter has (2^*n*^ + 1) points, where *n* is incremented by 1, until the second to largest subset is reached. The last subset contains all {*U*^(*s*)^} points. Each successively larger subset at the *n*-th level has one more point roughly halfway between two successive points from the previous subset at the (*n* − 1)-th level, based on the sort order indexes. Data points are added such that each subset contains all points within the previous subset. The base subset has sufficient span of the data sample to allow the dimension of the search space and the Lagrange multipliers to be rapidly determined. For a sample of 1 million data points, one calculation in the base subset will be almost 1000 times faster compared to using all the data. A single calculation in each subset thereafter takes about twice as long as it took in the immediate previous subset. It is worth mentioning that sort ordering data is done only once, because all subsets observe the same original ordering of the sample dataset.

To move into the next larger subset, the ***λ*** parameters from the current subset are used as an initial condition. In cases where the true PDF does not have sharp features, all subsets beyond the first consume a tiny fraction of the total computational cost because the expansion readily converges on the first subset. For cases where the true PDF has sharp features, larger subsets are important to refine the estimates inferred from lower level subsets. To capture sharp features requires a greater number of Lagrange multipliers, which slows down the calculation because greater accuracy is gained as more rapidly oscillating orthogonal functions are incorporated in the larger subsets. Although the hierarchical augmentation of data is not required to implement the algorithm, it renders the computational cost to an acceptable level for high throughput applications.

## Key result: Universal scoring function

To quantify the typical fluctuations that are expected in SURD, extensive numerical experiments were conducted. Using a robust uniform random number generator [59], a sample of *N* random numbers is generated on [0,1]. At least 100,000 samples are made per *N*, with millions of samples for small *N*. The probability given by Eq (3) for locating *U*^(*s*)^ in a sample is multiplied together to define a product measure given as

$$P_{L}\left(\{U^{\left(s\right)}\}\right)=\prod_{s=1}^{N}p_{s}(U^{\left(s\right)}|N)$$ (7)

The natural logarithm of Eq (7) is taken to transform a product of terms into a sum of terms. Dividing this sum by *N* defines an average for the entire sample, which quantifies single order statistics for the {*U*^(*s*)^} configuration. Numerical experiments reveal that the expectation of these mean values over an ensemble of samples is accurately described as a linear function of ln(*N*) such that

$$E\;\left[\frac{1}{N}ln(P_{L})\right]=\frac{1}{2}\left(\ln\left(N\right)-\frac{4}{5}\right).$$ (8)

Subtracting the systematic ln(*N*) dependence from each sample, a modified quasi-log-likelihood metric is defined as

$$L\left(\{U^{\left(s\right)}\}\right)=\frac{1}{N}ln\left(P_{L}(\left\{U^{\left(s\right)}\right\})\right)-\frac{1}{2}ln(N).$$ (9)

The modified quasi-log-likelihood metric, *L*, is sensitive to small variations in ***λ*** that affect the shape of the trial PDF, which changes how {*U*^(*s*)^} is distributed on the interval [0,1]. Henceforth, this *L* metric will simply be referred to as log-likelihood with the qualifiers “*modified quasi”* dropped. The word quasi was used as a reminder that unlike normal maximum likelihood methods, the product function in Eq (7) has built in correlations due to the sorting. After we developed this approach, it came to our attention that the quasi-log-likelihood metric is similar to the Anderson-Darling Test [60] although the way we have approached the problem is different, and with different objectives.

**Fig 3:**
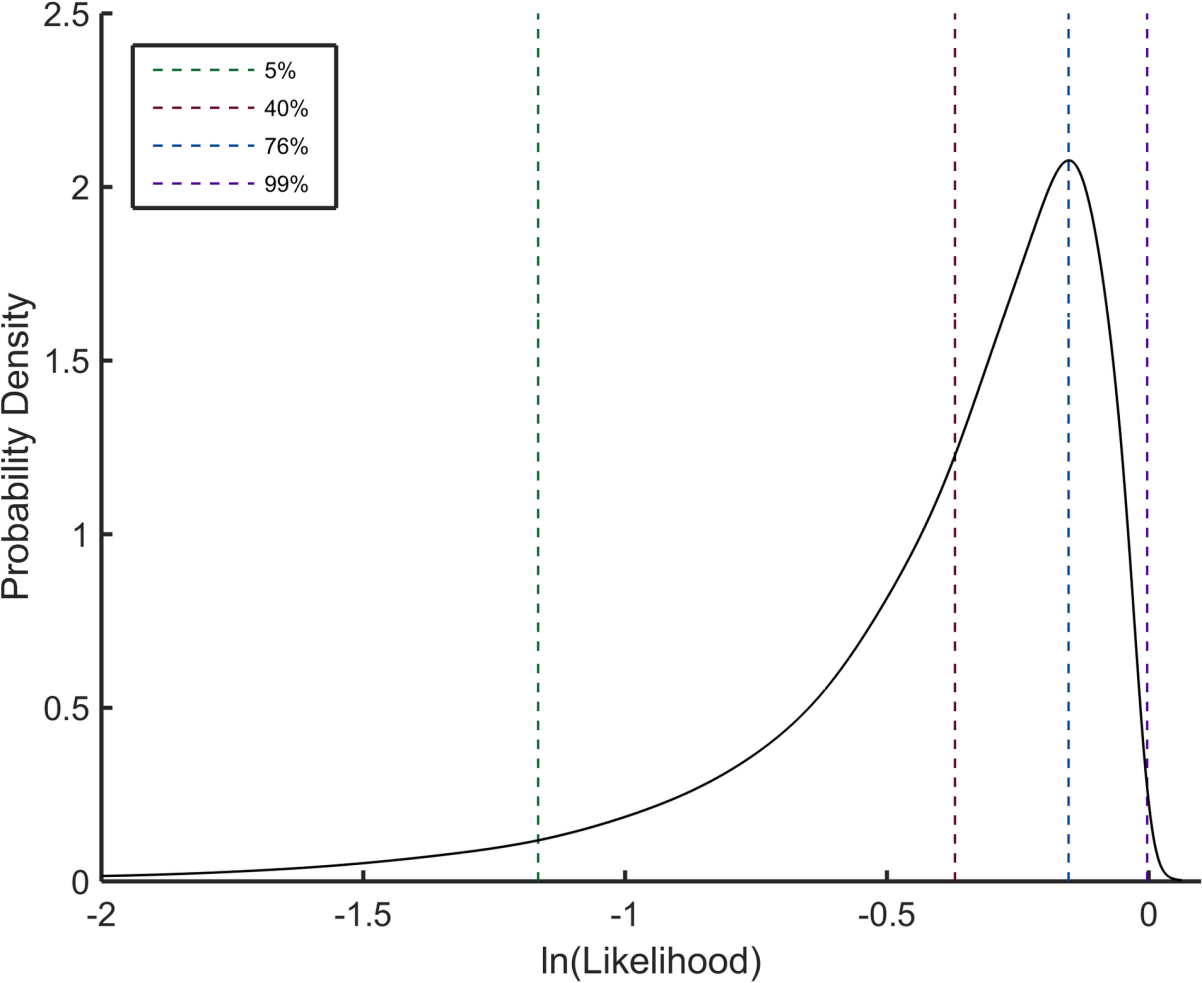
Probability density for SURD scoring function. The probability density for the log-likelihood score based on single order statistics for different sample sizes exhibits near sample size invariance. The solid black line is a consensus of all sample sizes combined, and the dashed lines show example levels of SURD coverage.

The average *L* that is calculated for SURD is −0.40 for any sample size. Interestingly, the PDF for the log-likelihood metric across different sample sizes exhibits data collapse, meaning the distribution is nearly sample size invariant as shown in Fig 3. Small variations between sample sizes do not lead to any practical concerns.

Therefore, we have combined log-likelihood metrics from different sample sizes ranging from 2^8^ to 2^20^ by factors of 2 to construct a universal scoring function. The maximum of the log-likelihood metric occurs when each *U*^(*s*)^ lands at the mode position of the s-th sorted value (i.e. the *u* where *p*_*s*_(*u*|*N*) is a maximum defines its mode). From numerical comparisons with least square error and binning statistics, the log-likelihood metric of Eq (9) provides the best sensitivity to discriminate between typical and atypical {*U*^(*s*)^} configurations, and it affords rapid evaluation.

An important issue is to model the PDF at the boundaries of the sampled data correctly. To ensure proper limits are satisfied requires augmenting a penalty term to the scoring function. The augmented penalty term was found to be essential only for distributions with heavy tails, and its functional form is not critically important. We use a penalty that is subtracted from *L* based on deviations of {*U*^(*s*)^} from their mean near the boundaries. Specifically,

$$penalty=ln\left[1+\frac{0.1}{p}\sum_{i=1}^{p}\left|U^{\left(i\right)}-\frac{i}{N+1}\right|+\frac{0.1}{p}\sum_{j=N-p}^{N}\left|U^{\left(j\right)}-\frac{j}{N+1}\right|\right]$$ (10)

Here *p* = ⌊*N* * 0.005⌋, meaning 1% of the data (0.5% at the left and right boundaries) is considered. This penalty has virtually no effect on good solutions, and for these good solutions the penalty term can be removed without inducing any negative consequence. The penalty term is necessary only at the start of a random search because it guides the random search method strongly when the ***λ*** parameters are far from converged.

It is worth noting that when hierarchical augmentation is employed, Eq (9) needs to be modified for each subset. Within the *p*-th subset, *N*_*p*_ is the number of samples being used. The first term that needs to be evaluated in Eq (9) per iteration is a self-averaging quantity. As such, the required modification to track the log-likelihood is given as:

$$L\;\left({\left\{U^{\left(s\right)}\right\}}_{p}\right)=\frac{1}{N_{p}}ln\left(P_{L}({\left\{U^{\left(s\right)}\right\}}_{p})\right)-\frac{1}{2}ln(N)$$ (11)

where {*U*^(*s*)^}_*p*_ is *N*_*p*_ data points uniformly spaced across the sort ordered indices.

Initially, the main task is to maximize the log-likelihood function. But at some point, maximizing log-likelihood makes the {*U*^(*s*)^} less random because it becomes atypically perfect. In other words, maximizing this function will lead to over fitting the data. Thus, *maximizing the log-likelihood is not an appropriate goal*, but rather, the objective is to set an acceptable range on the scoring function that represents typical outcomes. With the probability density of log-likelihoods for a sample of *N* data points determined, a universal confidence window can be defined. For example, to arrive at a confidence of 90%, the area under the curve of the PDF shown in Fig 3 for both the lower and upper tails of the scoring function can be cutoff at the 5% level. Hence, the cumulative distribution of log-likelihoods for SURD would be marked between 5% and 95%.

In practice, reaching a target score terminates the process of optimizing the Lagrange multipliers. We refer to this target score as the SURD coverage, defined as the area under the curve represented by the log-likelihood score PDF. For example, a log-likelihood value of about -0.15 represents approximately 77% SURD coverage, and this target corresponds to the most probable value according to the peak density in Fig 3. At 100% SURD coverage, the transformed data points on [0,1] will be uniformly spaced, but this “optimal” PDF is not the goal. Otherwise, random fluctuations within an input sample-A will almost surely be over-fit, meaning the expected level of fluctuations will be suppressed. As such, applying the CDF for sample-A (at 100% SURD coverage) on another random sample-B will likely amplify fluctuations compared to typical levels. In other words, our goal is to observe typical fluctuations within any SURD sample, and the universal log-likelihood function quantifies this. The most probable value of the universal log-likelihood PDF is where the most frequent log-likelihood scores for SURD fall, which defines a target score that is considerably lower than the maximum possible score corresponding to near perfect uniform spacing.

As argued, excellent results are achieved by enforcing a SURD coverage target of 77%. However, we do not need to reject lower scores that do not reach this target since a wide range of lower scores make up a substantial portion of typical fluctuations. We should only consider rejecting solutions with very low scores corresponding to a 5% SURD coverage or less. After much experimentation, and taking into consideration computational cost tradeoffs, it was found that a target log-likelihood value of −0.37 with approximately 40% SURD coverage provides a reasonable target value. However, should the program fail to hit the target of 40% SURD coverage, the final solution is not rejected if it achieves at least 5%. This final step corresponds to step 9 in Fig 1, and is only invoked when all attempts to hit the target value have been unsuccessful.

By dropping from a target of 77% to 40% SURD coverage, computing time was improved by worthwhile factors ranging from 10% to 50% speed increases depending on feature sharpness in the true PDF. Dropping below 40% SURD coverage does not yield substantive reduction in compute time. Moreover, when SURD coverage from a target value is in the range from 20% to 60% similar visual results are obtained in the PDF estimates and the SQR-plots. Qualitatively we find that as the SURD coverage target is increased gradually, the SQR-plot shows similar random pattern but with a commensurate gradual decrease in amplitude to a point that is atypically small as SURD coverage approaches 100%. Therefore, 40% is invoked as the default SURD coverage, and, although an arbitrary number, it falls within a non-sensitive zone that consistently produces good quality solutions in an efficient manner for all cases considered in this study.

## Benchmark examples and discussion

The nonparametric MEM for PDF estimation is assessed on five different types of test-distributions. In all cases, random samples are first analyzed using *default settings*. Default settings are designed for high throughput applications where it is assumed there is no knowledge of the form of the distributions to be uncovered. In particular, default settings assume the PDF has tails that may extend to ± infinity. When the distribution has sharp boundaries the nonparametric MEM estimates using default settings are of good quality in stark contrast to KDE. However, because additional information can be used when available (e.g. known boundary conditions or symmetries) some benchmark cases highlight the features of this method.

### 4.1 Generating and assessing results

The scope of the testing involves four different sample sizes, with *N* = 2^8^, 2^12^, 2^16^, 2^20^, where the factors of 2 are considered only for convenience in presenting results. Performance does not depend on any special value of *N*. Due to greater statistical resolution inherent in larger sample sizes, the quality of the PDF estimate is monitored as *N* increases. For each sample size, four independently drawn samples are randomly generated from the true PDF to produce a total of sixteen sample sets. Five possible model PDF solutions are calculated per input sample, yielding 20 model PDF solutions per sample size. The procedure employed to generate data samples is the same for all test distributions. Given a test-distribution defined by its PDF, *p*(*v*), the corresponding quantile function, *Q*(*u*), is constructed. A set of random samples {*V*_*k*_} are generated through the transformation *V*_*k*_ = *Q*(*r*_*k*_), where *r*_*k*_ is a uniform random number on [0,1] using a high quality random number generator [59].

For a given data sample, the central model PDF among 5 possible solutions is identified as the PDF estimate, having a minimum total pairwise squares error between it and the other four PDF models. The standard deviation for the differences between the alternate PDF models with respect to the central model (the estimate) can be used to report error bars on the PDF estimate (not shown here). An interesting question is how similar are the various PDF estimates (one for each sample) to each other? While PDF estimates for different samples can differ from the true PDF due to finite size fluctuations inherent within a sample, the method attempts to resist over fitting to sampling fluctuations. To see how well the method works for each test-distribution, the PDF-estimate is shown for each of the four input samples for each of the different sample sizes, and they are compared to the true PDF used to generate the samples. In addition, for each PDF estimate its corresponding SQR-plot is shown.

The SQR-plots provide an intuitive visual assessment of important characteristics concerning the quality of the PDF-estimate. In particular, Fig 2 shows two hallmark features of SURD data, which we will use as a baseline for comparison. As already noted, the scaled fluctuations typically fit comfortably within the gray ovals in Fig 2, independent of sample size. These fluctuations characterize random error found from one sample to the next. Should these fluctuations have small amplitudes about the zero line, spanning very little area of the oval, this would suggest a PDF that is over fit to the data, whereas fluctuations that are near the edges of the oval or fall outside of the oval, would indicate an under fit.

Another feature about the SQR-plots for SURD in Fig 2 is that the differences in fluctuations between the four independently generated sample sets are also random. In contrast to random errors, regular patterns in fluctuations across all data samples would indicate systematic errors. Regardless of the basis functions used, when a truncation is employed in a series expansion, systematic error in representing the true PDF will be incurred that is not related to statistical resolution. Adding Lagrange multipliers is the only way to reduce this systematic error. However, reducing this systematic error may also reduce the random error, thus over fitting to data. The PDF-estimation algorithm simply strives to reduce the error averaged over all data samples, without distinguishing between systematic and random. In difficult cases, some regions may be under fitted, while other regions compensate for these discrepancies by being over fitted in order to reach the target SURD coverage with a smaller number of Lagrange multipliers. Thus, the SQR-plot is a valuable diagnostic tool for evaluating the nature of the residual error. The non-random regular patterns observed can be used for making corrections. It is worth mentioning that the SQR-plot can be applied to any PDF estimate (based on any method) after the PDF is integrated to obtain a CDF.

For each test-distribution, four statistical metrics are tabulated to summarize the quality of the PDF estimate for each independent sample drawn from the true PDF. The metrics include the *p*-value for the two-sample Kolmogorov-Smirnov (KS) test [61] that compares the input sample to a random sample generated from the estimated PDF. A *p*-value that is lower than 0.05 suggests the generated sample is different from the input sample, although 5% of the time samples from the true PDF will return a p-value of 0.05 or less. The second metric is the Kullback–Leibler distance (KL-distance) [62, 63]. The *Figure of Merit* (*FOM*) defined in the methods section is a third metric. When *FOM* > 0 there is high confidence that the input sample reflects a typical outcome from the estimated PDF. The SURD coverage is reported as the fourth metric. In addition, the number of Lagrange multipliers used to determine the PDF is reported, which highlights variations between PDF estimates.

With the exception of the KL-distance, all evaluation metrics are calculated without knowing the true population distribution. About 85% of the time all metrics unanimously indicate at least a fair solution. These results are respectable considering that the test-distributions pose diverse challenges. In two cases the default KDE implementation in MATLAB failed, and in two other cases the KDE results were notably worse visually. In particular, KDE is noted to be problematic in describing heavy tails and sharp boundaries. It is our experience that KDE typically requires human experimentation to obtain satisfactory results for cases when it works, and in some cases, such as the gamma distribution, it simply fails.

### 4.2 Gamma distribution

**Fig 4:**
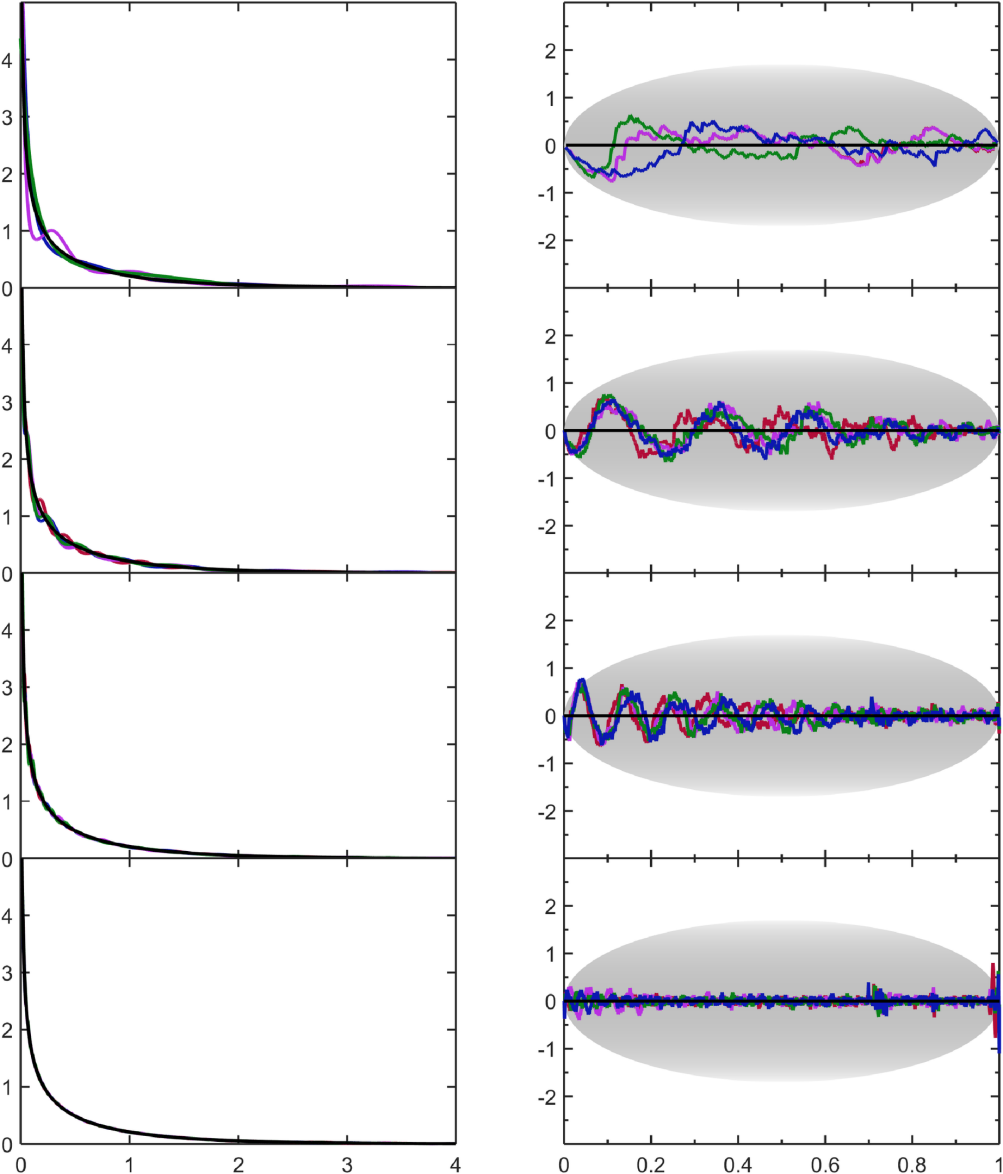
Assesment of results for the gamma distribution. The left column shows the estimated PDF based on four samples colored as green, red, blue and magenta per sample size. Results for different sample sizes are also shown ranging from *N* = 2^8^, 2^12^, 2^16^, 2^20^ moving from the top to bottom rows. These estimates are compared with the true population PDF shown as a black line. The right column shows the corresponding SQR-plots across respective rows.

Here, $p(v)=\frac{1}{\sqrt{\pi v}}e^{-v}$ on (0,∞). This special case of a gamma distribution contains a square-root singularity, which requires no special treatment with the approach taken here. Despite the divergence when *v* → 0, the functional form of Eq (2) provides a good representation of the sampled data. The PDF estimates are shown in Fig 4 where no boundary conditions are specified (default case). The *FOM* metrics and the visual appearance of the SQR-plots indicate the PDF estimates have systematic errors. When the number of Lagrange multipliers is small the PDF estimates incur systematic error because the true PDF cannot be represented by a truncated series of expansions that has not yet converged. However, as sample size increases more expansions are used, and this relegates the systematic wiggles to appear on a finer length scale. For the largest sample size, systematic error is not visible in the PDF estimate, but the SQR-plot shows deviations with overall smaller amplitude than the expected typical fluctuations. This test case highlights a general result that errors due to random fluctuations from finite size samples and systematic errors due to inadequate functional representations compete with one another during the minimization of the log-likelihood scoring function.

We made no effort to account for this in the current implementation. In future improvements, an additional penalty term could be included to separate out systematic and random errors given that outliers in the SQR-plots are easy to identify and quantify objectively. Nevertheless, the current implementation already provides a robust automated process that yields good results as summarized in Table 1 when considering the balance between automation, speed and accuracy. In contrast, a typical KDE approach fails to capture the singularity altogether. In addition, better results are obtained with the current approach if a priori information is used about the data being only positive or zero. Importantly, as seen in Table 1 more Lagrange multipliers are needed to model the singularity with an accuracy that is commensurate to statistical resolution as sample size increases.

**Table 1:** Characteristics of PDF estimates for the gamma distribution.

Sample Size	p-value	KL distance	Figure of Merit	SURD coverage	Lagrange Multipliers
256	0.59 0.59 0.60 0.60	9.2E-3 1.6E-2 1.6E-2 2.4E-2	0.90 0.89 0.90 0.88	0.40 0.40 0.40 0.41	9 9 9 13
4096	0.31 0.33 0.38 0.41	1.1E-2 1.0E-2 1.1E-2 1.1E-2	0.59 0.64 -0.58 0.65	0.40 0.40 0.40 0.41	33 29 31 29
65536	0.17 0.17 0.17 0.18	1.0E-2 1.0E-2 9.9E-3 9.6E-3	-0.02 -0.23 0.02 -0.15	0.41 0.43 0.40 0.40	69 77 77 81
1048576	0.28 0.36 0.44 0.46	9.9E-3 9.9E-3 9.9E-3 9.8E-3	-0.41 -0.37 0.30 0.15	0.18 0.17 0.34 0.28	239 233 213 289

### 4.3 Sum of two Gaussian distributions

Here, $p(v)=\frac{7}{10}N(v|\mu_{1}=5,\sigma_{1}=3)+\frac{3}{10}N\left(v|\mu_{2}=0,\sigma_{2}=\frac{1}{2}\right)$ on (−∞,∞) where N(v|μ,σ) denotes a Gaussian distribution with mean *μ* and standard deviation *σ*. This example was contrived so that the PDF cannot be simply expressed as a single exponential in accordance with Eq (2). The less probable population appears as a small shoulder protruding out from the side of a broad Gaussian form. As such, a large number of Lagrange multipliers will be necessary to match the functional form of the true PDF. By design, small sample sizes do not have the statistical resolution necessary to discern the difference between a sample fluctuation and the presence of a sharp Gaussian shoulder hiding within the broader Gaussian.

The results in Table 2 inform us that the number of Lagrange multipliers increase as sample size is increased. This happens because there are features that cannot be fully resolved with small number of sample points. It is also clear by comparing Table 2 to Table 1 that the gamma distribution with the singularity at the origin is difficult to recover and some metrics show that some PDF estimates are of poor quality (but fortunately, they are still useful). For this two-Gaussian case, Table 2 shows that all PDF models generated are excellent. When the test-distribution does not have extreme features (singularities, heavy tails or discontinuities) the various metrics that characterize the quality of the PDF estimates have been found to be excellent.

**Table 2:** Characteristics of PDF estimates for the bimodal distribution described by a binary mixture of two Gaussian distributions.

Sample Size	p-value	KL distance	Figure of Merit	SURD coverage	Lagrange Multipliers
256	0.50 0.51 0.54 0.62	1.3E-2 1.3E-2 1.4E-2 1.3E-2	0.90 0.89 0.90 0.87	0.43 0.46 0.40 0.42	3 3 4 3
4096	0.46 0.51 0.54 0.55	5.7E-3 5.2E-3 7.8E-3 9.0E-3	0.92 0.92 0.91 0.91	0.41 0.41 0.42 0.40	17 17 13 13
65536	0.51 0.53 0.55 0.62	5.2E-3 5.8E-3 6.1E-3 7.5E-3	0.89 0.87 0.89 0.84	0.40 0.40 0.40 0.42	33 33 33 29
1048576	0.43 0.50 0.51 0.57	6.7E-4 7.2E-3 5.1E-3 5.0E-3	0.90 0.90 0.89 0.90	0.40 0.40 0.40 0.40	53 49 47 47

**Fig 5:**
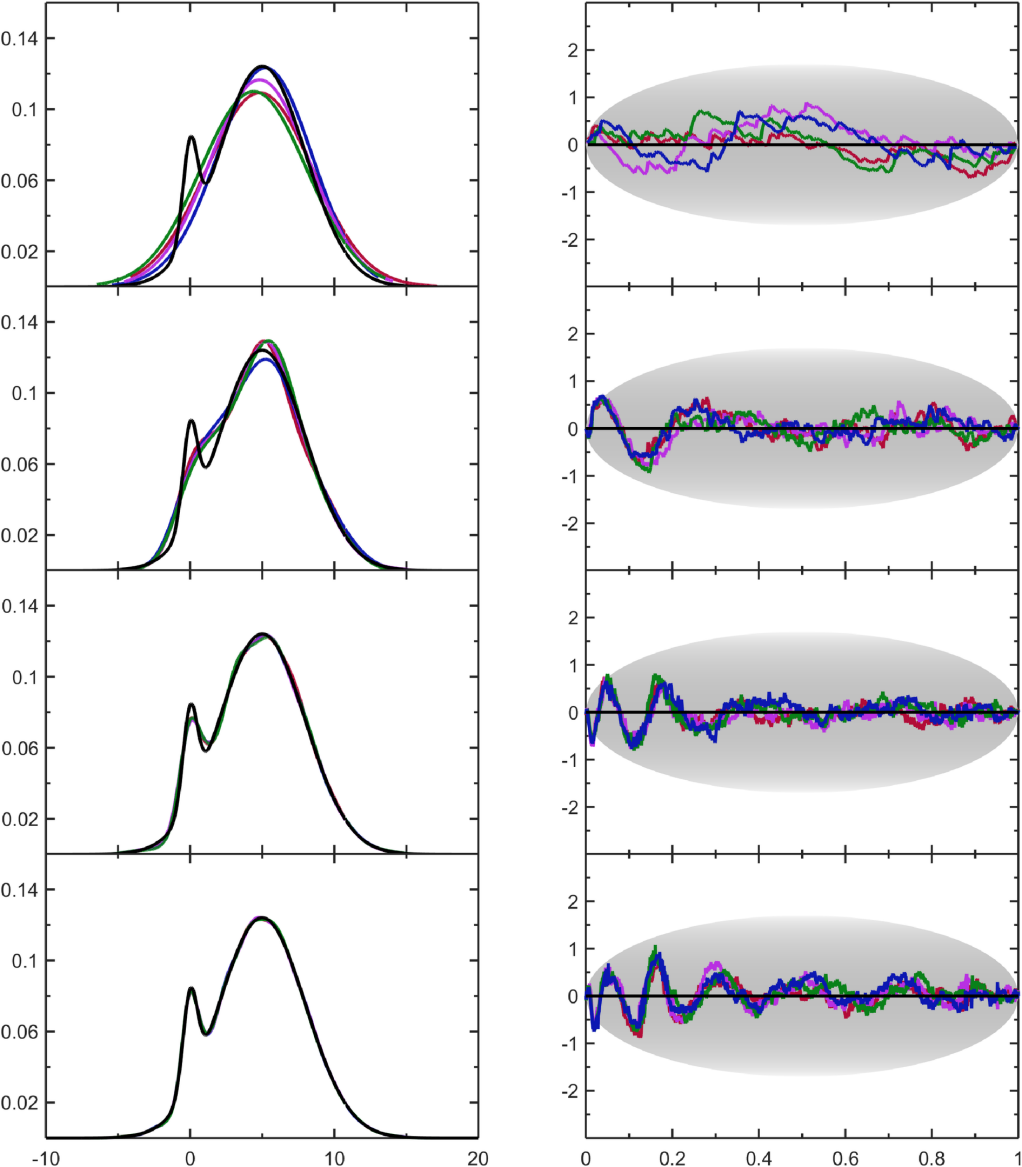
Assesment of results for the sum of two Gaussian distributions. The data format and coloring is the same as Fig 4.

The quality of the PDF estimate improves with sample size as shown in Fig 5. The small shoulder is essentially ignored at a sample of size of 256 data points because the method is resistant to over-fitting data. Similar to the gamma distribution results, systematic error is present in the form of oscillations in the SQR-plot for all but the smallest sample sizes. Interestingly, this is the only case among those considered here that KDE had better performance (quality and speed combined), albeit the protruding shoulder is subject to being over fit at small sample size by KDE, which is easy to do when the peak is a priori known to be present in the true PDF.

### 4.4 Five fingers distribution

Here, $p(v)=w\;\sum_{k=1}^{5}\frac{1}{5}N(v|\mu_{k}=\frac{2k-1}{10},\sigma =\frac{1}{100})+(1-w)$ on [0,1] where N(v|μ,σ) denotes a Gaussian distribution with mean *μ* and standard deviation *σ*. Five sharp Gaussian distributions are added to a uniform distribution. The case shown here corresponds to *w* = 0.5. Due to the sharpness of all five Gaussian distributions it is virtually exact to consider the domain to be on the range [0,1] without cutting off the tails of the Gaussian distributions and without having to renormalize the PDF. The five fingers distribution demonstrates how the nonparametric MEM resolves the Gaussian shaped fingers better as statistical resolution is increased with greater sample size. As seen in Table 3 more Lagrange multipliers are needed to resolve features as sample size increases. In this case, the algorithm is beginning to reach saturation in its ability to improve, at least with respect to the p-value metric. This is likely because the model PDF has shallow wiggles near the sharp peaks. These wiggles come from systematic error in the expansion.

**Table 3:** Characteristics of PDF estimates for a five fingers distribution with a 0.5 weight.

Sample Size	p-value	KL distance	Figure of Merit	SURD coverage	Lagrange Multipliers
256	0.33 0.37 0.39 0.42	3.4E-1 4.1E-1 4.7E-1 4.1E-1	0.89 0.89 0.88 0.90	0.42 0.41 0.41 0.43	15 11 15 7
4096	0.17 0.20 0.20 0.22	1.1E-1 9.5E-2 1.1E-1 1.3E-1	0.62 0.62 0.49 0.57	0.40 0.40 0.40 0.40	35 37 35 35
65536	0.06 0.07 0.07 0.08	1.6E-2 1.5E-2 1.5E-2 1.6E-2	0.55 0.43 0.65 0.62	0.40 0.41 0.40 0.41	51 53 53 51
1048576	0.02 0.03 0.05 0.06	2.4E-3 2.2E-3 2.1E-3 2.4E-3	0.04 0.32 0.87 0.84	0.42 0.42 0.42 0.42	99 101 97 101

**Fig 6:**
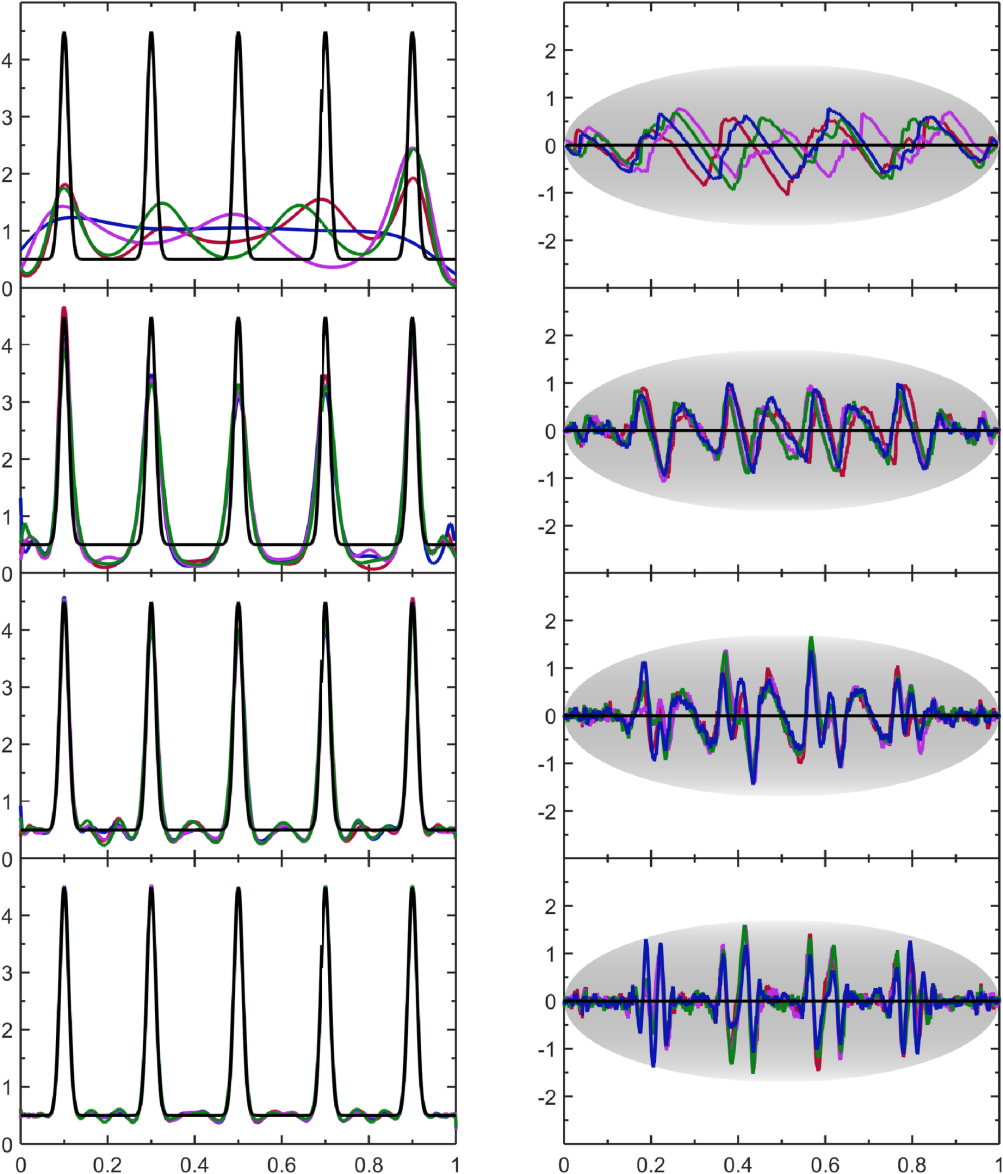
Assesment of results for the 5-fingers perturbing the uniform distribution with a 0.5 weight. The data format and coloring is the same as Fig 4.

Fig 6 shows that the five fingers are visually described well. In contrast, poor results were obtained for the five-finger-distributions using KDE, especially for large sample sizes. Importantly, at a sample size of 256 points the nonparametric MEM cannot discern the fingers compared to random sample fluctuations, which means the estimated PDF is close to a uniform. The oscillations seen in the SQR-plots in Fig 6 exemplify the compromise made between systematic and random errors. Indeed, the PDF estimates have an appearance that is reminiscent of using a truncated Fourier series to approximate a periodic waveform with sharp features.

### 4.5 Cauchy distribution

Here, $p(v)=\frac{b}{\pi (v^{2}+b^{2})}$ on (−∞,∞) where $b=\frac{1}{2}$ is the classic Cauchy distribution, which has a heavy tail and models extreme statistics in the sense that the second moment, 〈*v*^2^〉, and the standard deviation do not exist. Distributions with extreme statistics are generally challenging for a KDE approach, unless the tails are severely cut in advance, but this creates error in the estimate. It turns out that the MEM presented here also requires censor windowing for it to provide stable results. As described in section 2.2, the employed default censor window on [*a*,*b*] are assigned limits given by *a* = *Q*_25_ − 7(*Q*_75_ − *Q*_25_) and *b* = *Q*_75_ + 7(*Q*_75_ − *Q*_25_) and this range is sufficiently restrictive to handle this Cauchy distribution without user-defined overrides. All calculations are stable and the results within the censor window do not depend on the data that is filtered out. This censor windowing boundary rule in terms of quantiles is applied to any input data for which the user has no knowledge as the default setting.

**Fig 7:**
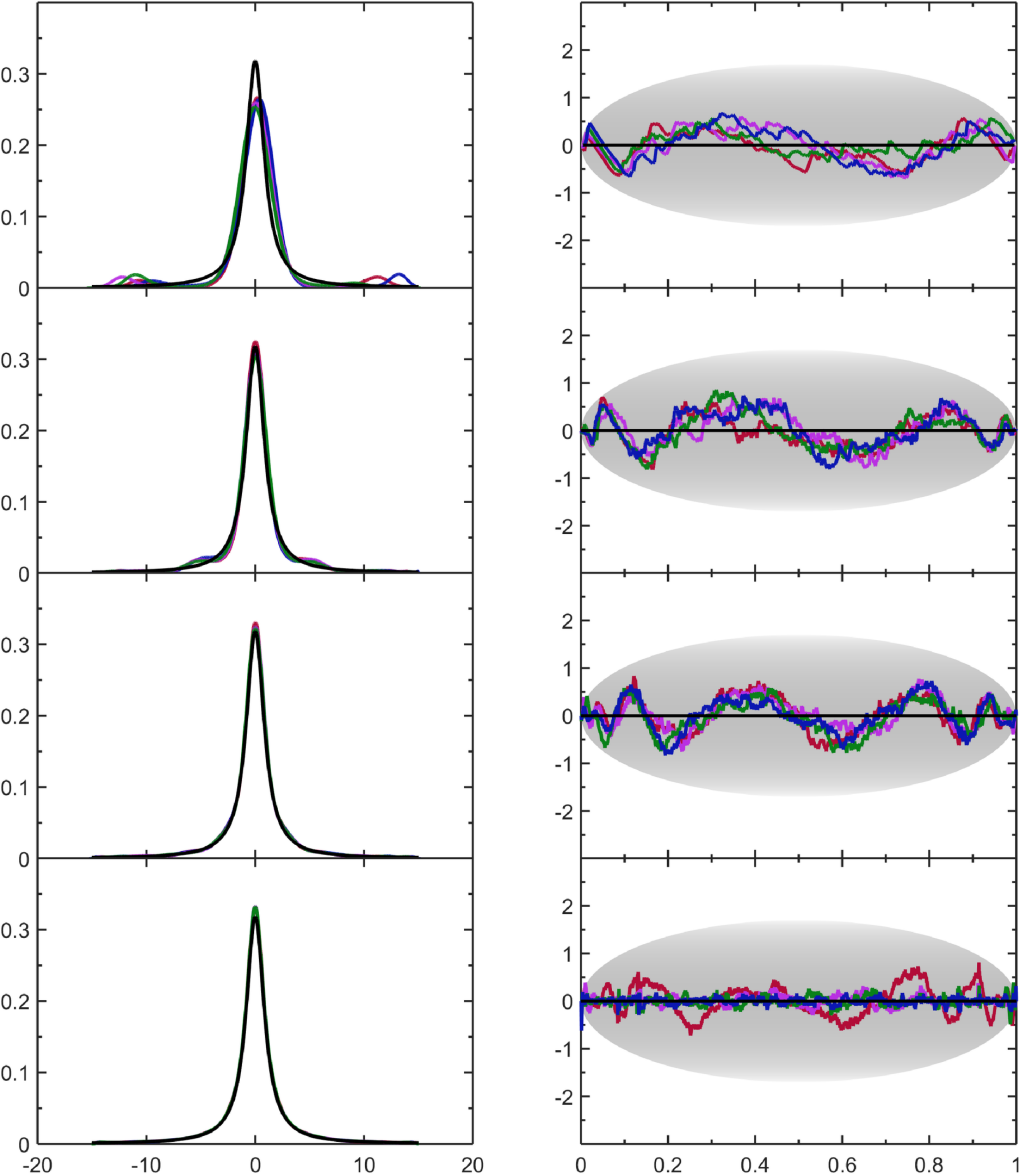
Assesment of results for the Cauchy distribution. The data format and coloring is the same as Fig 4.

In Fig 7, it is clear that increasing the number of samples provides a better representative of the Cauchy distribution despite more outliers. With the exception of some wiggles that appear in the tails of the PDF estimates at small sample sizes, the nonparametric MEM provides robust statistical inference. For the *N* = 256 sample size, Fig 7 shows that the large bumps in the tails in the PDF estimate decay to zero further out. Although the bumps in the tails at small sample size certainly do not look good, it is consistent with SURD at the set target value. The bumps in the tails are reflecting outliers. It is worth noting that by enforcing some smoothness conditions in the tails it is possible to obtain a model PDF that looks closer to the true PDF (e.g. removal of bumps) when the number of samples is small, but inevitably for other cases where the true PDF has a different form, extra smoothness conditions will generally yield worse results in the sense that the true PDF form is missed. The best compromise we found is not to smooth at all, even though smoothing remains a user-defined option following past work [47], which is useful if the there is a priori justification.

### 4.6 Discontinuous distribution

Here, the true PDF is defined as a discontinuous distribution on the interval [0,1], given as:

$$p(v)=\left\{ \begin{array}{l} \;4/5,\;v<0.3\;or\;v>0.8\\ 1,\;v>0.4\;and\;v<0.5\\ \;5/4,\;otherwise \end{array}\right.$$

**Fig 8:**
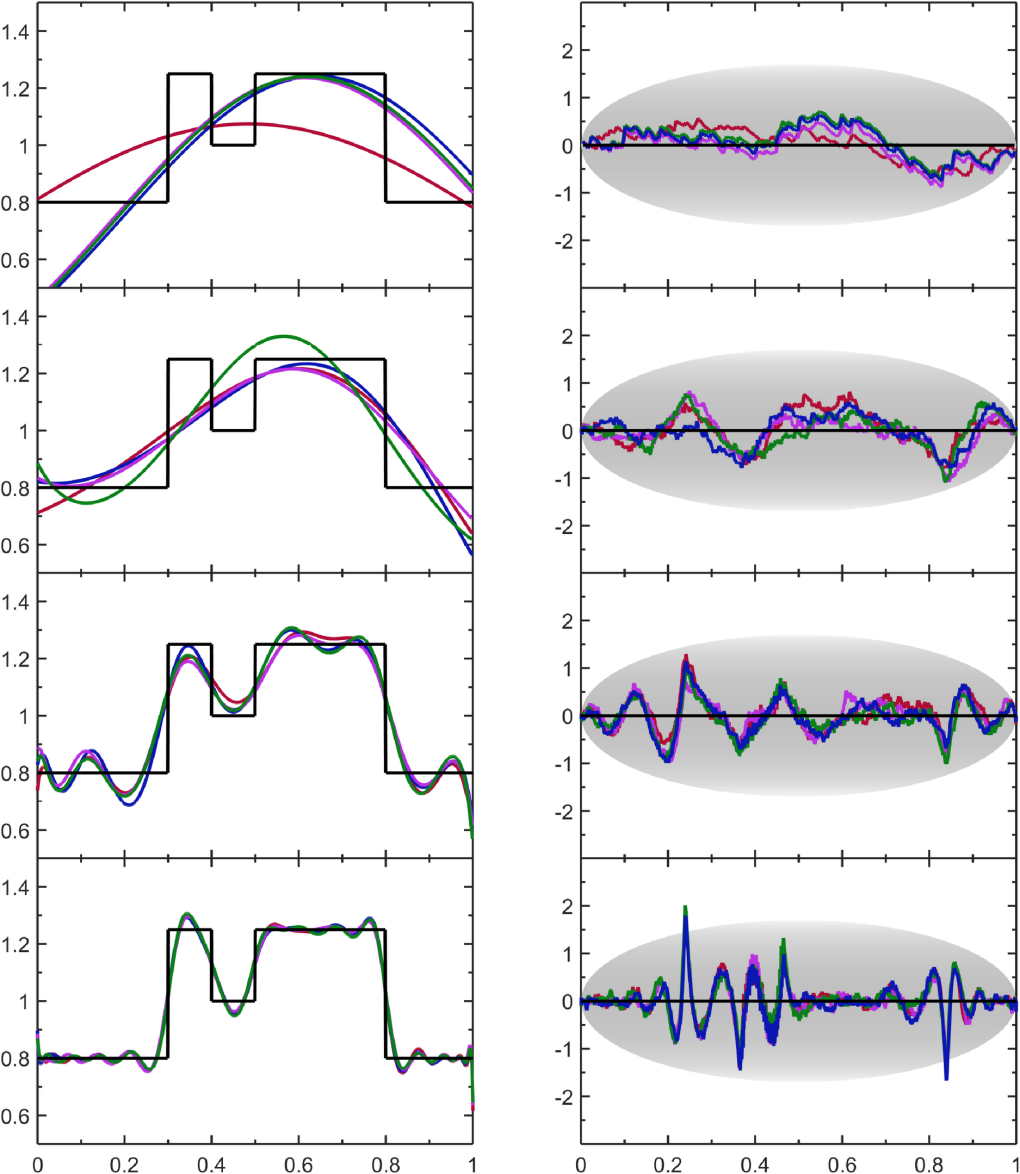
Assesment of results for the discontinuous distribution. The data format and coloring is the same as Fig 4.

The expansion in Eq (2) can approximate a discontinuous distribution within the accuracy of statistical resolution. For small sample sizes, infinitely sharp discontinuous features are replaced by broad smooth differentiable continuous curves that make no attempt to follow the abrupt changes. As sample size increases the discontinuous features are automatically modeled more accurately as statistical resolution increases. For sharp edges to come into focus the number of Lagrange multipliers must be increased. Fig 8 shows the PDF models and the SQR-plots. Comparison of the PDF models with the true PDF shows that the sharp discontinuities are captured, although more wiggles are found along the flat plateaus than what KDE produces. The large systematic errors observed in the SQR-plots reflect rounding of the discontinuities.

For small samples, results from this nonparametric MEM are similar to those obtained by a KDE method. At large sample sizes, KDE methods provide smoother results than the MEM used here due to systematic error as discussed above. In this example, the user can invoke the SQR-plots to elucidate where errors occur. By inspection of Fig 8, it can be seen that positive peaks and negative troughs in SQR-plots respectively reflect over and under estimates of probability density relative to the true PDF. Furthermore, for the largest sample size, there are peaks that extend beyond the grey envelope of expected maximum values, indicating these fluctuations are highly atypical at this resolution, revealing the presence of systematic error at discontinuities reminiscent of what occurs with Fourier analysis.

Given that systematic error in the PDF models is visually distinct from random noise within the SQR-plot, a fruitful direction we have explored is to employ signal processing methods to extract systematic deviations from the SQR-plot and use them to correct the PDF estimate. However, this signal processing method presents only one possible route for improving the current algorithm as a post-solution correction. Another approach to suppress systematic error while leaving typical random errors intact is to add penalty terms to the scoring function when the SQR-plot of a trial CDF deviates far from the expected random behavior (i.e. outlier detection). For example, outlier detection can be based on how far a fluctuation exceeds the boundary of the expected typical range of fluctuation shown as an oval in the SQR-plots. An outlier penalty term would allow the regions that are currently being over fitted to maintain typical levels of fluctuations as the need to compensate for systematic errors is suppressed. As a consequence, more expansion terms are likely to be added to reduce atypical fluctuations within the regions currently being under fitted. Thus, many fruitful directions are possible to improve upon the current algorithm in future work. Fortunately, the deficiencies that appear in these difficult examples are still tolerable and are straightforwardly detectable using objective measures.

### 4.7 Performance characteristics

**Fig 9:**
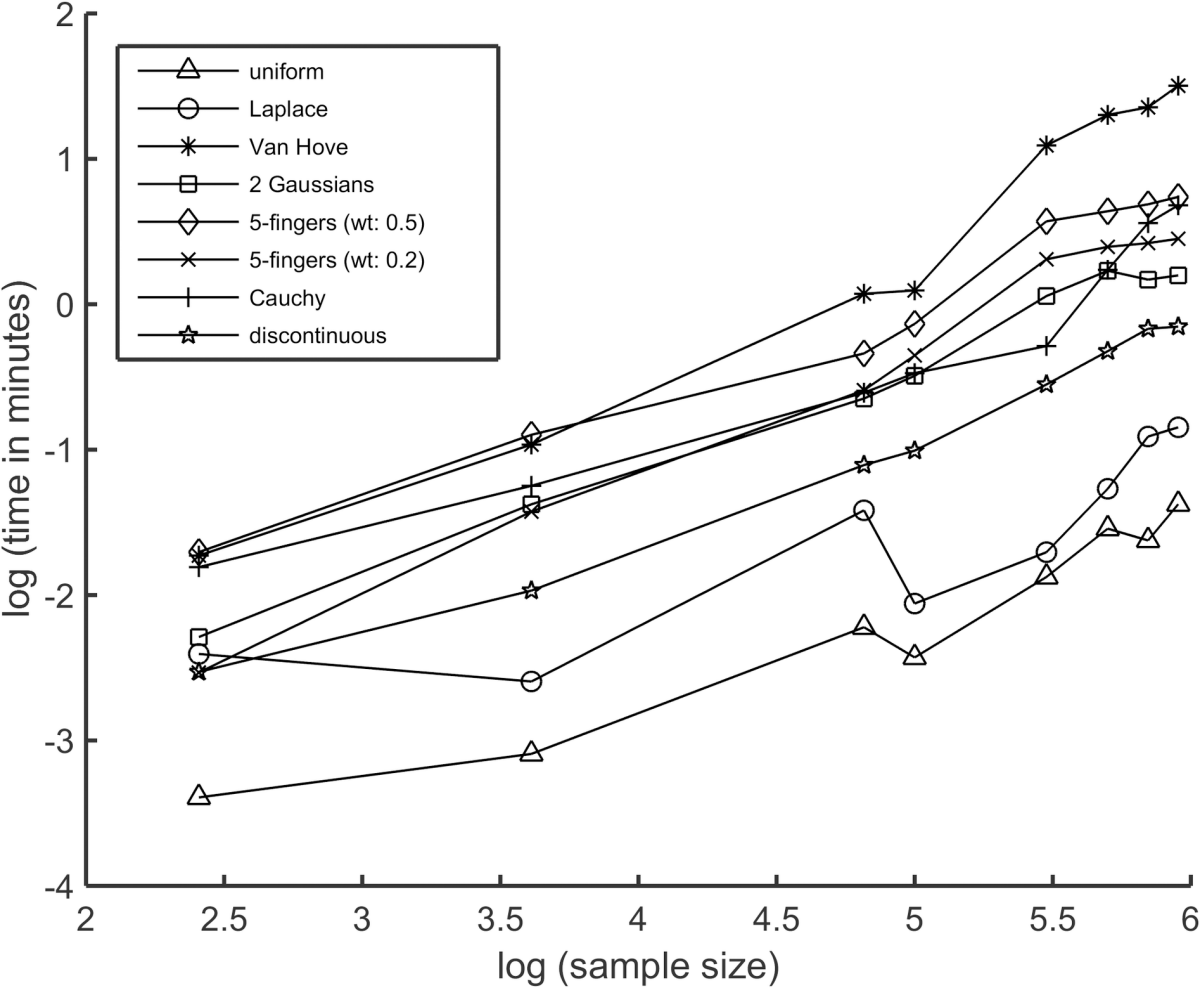
Mean CPU time. On a log10-log10 plot, the average CPU time to calculate a model PDF as a function of sample size for all distributions considered here in addition to a few others. The averaging was over 100 distinct solutions per sample size.

Hierarchical augmentation of the sampled data makes the cost of calculations relatively low. PDF models can be calculated for a sample containing 2^20^ data points in well under 1 minute for distributions requiring few Lagrange multipliers, such as uniform, Laplace, and Gaussian. The basic timing of the current implementation is shown in Fig 9 for the test-distributions described here in addition to a few other distributions. All tests were run on a high-performance computing cluster with dual Intel Xeon 2.93 GHz 6-core processors. However, it is noted that the code is not parallelized and better performance times is typical using modern laptops. For difficult test-distribution studied here, a model PDF for sample sizes of 25,000 data points typically take less than 1 minute. Because difficult distributions with many fine or sharp features need a large number of Lagrange multipliers at high statistical resolution, sample sizes of more than 25,000 data points are required to justify using a large number of Lagrange multipliers. For sample sizes of less than 1000 data points, poor statistical resolution prevents PDF estimates from having many Lagrange multipliers, which keeps computational cost minimal and the estimates smooth. Compared to KDE on difficult cases where KDE succeeded, the presented MEM can be more than an order of magnitude slower, but KDE results were generally of lower quality unless considerable effort was spent fiddling with non-default settings. It is worth mentioning that the nonparametric MEM presented here has not been optimized to the same level as highly efficient KDE methods with decades of development. With considerable room for further optimization the nonparametric MEM introduced here is likely to become more competitive in computing times while resolving the troubling areas that inflict KDE.

## Conclusions

In the modern era of high throughput data analytics, it is imperative to have a data-driven automated method to obtain accurate PDF estimates without human subjectivity or involvement in the process. To satisfy these needs we developed a nonparametric maximum entropy method to estimate a PDF for a sample of random data where little information about the data is known. It is only required that the random variables are continuous and that the observations are, or can be approximated as, independently and identically distributed. The functional form for the PDF is an exponential of a series expansion over a set of orthogonal basis functions. A random search method is used to determine the expansion coefficients using a trial CDF that transforms sampled random data onto the interval [0,1]. These new random variables, {*U*_*k*_}, are assessed using a scoring function based on a log-likelihood of single order statistics of *sampled uniform random data* (SURD). Importantly, the statistical properties of the scoring function are universal, being problem independent and sample size invariant, allowing atypical large or small fluctuations to be identified. As such, this approach is resistant to under and over fitting to sample fluctuations, and it is indifferent to the nature of the distribution. An ensemble of PDF models is generated from which the most typical result among them is selected as a PDF estimate for the population. Variation among PDF models from this ensemble places uncertainties on the PDF estimate, useful for risk assessment.

To help with benchmarking the results on test-distributions we introduced the scaled quantile residual plot (SQR-plot) and a figure of merit (FOM) to characterize how typical is the input data sample to a set of random samples generated from the estimated PDF. The presented method yields a PDF estimate that commensurately matches statistical inference with statistical resolution. Finally, the universal scoring function and diagnostic tools (SQR-plots and FOM) are applicable to other estimation methods. In particular, the universal scoring function (Fig 3) based on the quasi log-likelihood metric given in Eq (9) can serve as an objective function in optimization problems found in parametric and semiparametric methods, as well as KDE to optimize bin width selection. In future work, it would be natural to use this scoring function in model selection applications using Monte Carlo Markov Chains. With a slight modification of the current random search method we employ here, one can explore the high dimensional parameter space and look for multiple solution sets. Thus the approach we introduce here has many avenues by which it can be extended and applied ubiquitously across many fields.

## Supporting information

S1 Source Code A source.zip file contains the Java source code, a configuration file, a text file for the likelihood distribution that defines as the universal scoring function, and an example input file having random data sampled from a Gaussian distribution. (ZIP)
